# Predictors of non-recovery from fatigue and cognitive deficits after COVID-19: a prospective, longitudinal, population-based study

**DOI:** 10.1016/j.eclinm.2024.102456

**Published:** 2024-02-03

**Authors:** Tim J. Hartung, Thomas Bahmer, Irina Chaplinskaya-Sobol, Jürgen Deckert, Matthias Endres, Katrin Franzpötter, Johanna Geritz, Karl G. Haeusler, Grit Hein, Peter U. Heuschmann, Sina M. Pütz, Anna Horn, Thomas Keil, Michael Krawczak, Lilian Krist, Wolfgang Lieb, Corina Maetzler, Felipe A. Montellano, Caroline Morbach, Christian Neumann, Carolin Nürnberger, Anne-Kathrin Russ, Lena Schmidbauer, Sein Schmidt, Stefan Schreiber, Flo Steigerwald, Stefan Störk, Thomas Zoller, Walter Maetzler, Carsten Finke, NAPKON Study Group

**Affiliations:** a Charité - Universitätsmedizin Berlin, Department of Neurology and Experimental Neurology, Berlin, Germany; b Internal Medicine Department I, University Hospital Schleswig Holstein, Campus Kiel, Germany; c Airway Research Center North (ARCN), German Center for Lung Research (DZL), Grosshansdorf, Germany; d Department of Medical Informatics, University Medical Center Göttingen, Germany; e Klinik und Poliklinik für Psychiatrie, Psychosomatik und Psychotherapie, Uniklinik Würzburg, Germany; f Center for Stroke Research Berlin, Berlin, Germany; g ExcellenceCluster NeuroCure, Berlin, Germany; h German Center for Neurodegenerative Diseases (DZNE), Partner Site Berlin, Germany; i German Centre for Cardiovascular Research (DZHK), Partner Site Berlin, Germany; j Institute of Epidemiology, Kiel University, Germany; k Neurology Department, University Medical Center Schleswig-Holstein, Campus Kiel, Germany; l Department of Neurology, Universitätsklinikum Würzburg, Würzburg, Germany; m University of Würzburg, Institute of Clinical Epidemiology and Biometry, Würzburg, Germany; n University Hospital Würzburg, Institute for Medical Data Science, Würzburg, Germany; o University Hospital Würzburg, Clinical Trial Center, Würzburg, Germany; p University of Cologne, Faculty of Medicine and University Hospital Cologne, Department I of Internal Medicine, Center for Integrated Oncology Aachen Bonn Cologne Duesseldorf, Germany; q Charité - Universitätsmedizin Berlin, Institute of Social Medicine, Epidemiology and Health Economics, Berlin, Germany; r Institute of Medical Informatics and Statistics, Kiel University, University Medical Center Schleswig-Holstein Campus Kiel, Kiel, Germany; s University Hospital Würzburg, Department Clinical Research and Epidemiology, Comprehensive Heart Failure Center, Würzburg, Germany; t Germany University Hospital Würzburg, Department of Neurology, Würzburg, Germany; u University Hospital Würzburg, Department for Medicine I and Comprehensive Heart Failure Center, Germany; v Berlin Institute of Health at Charité – Universitätsmedizin Berlin, Clinical Study Center, Germany; w Department of Internal Medicine I, University Hospital Würzburg, Germany; x Charité – Universitätsmedizin Berlin, Corporate Member of Freie Universität Berlin and Humboldt-Universität zu Berlin, Department of Infectious Diseases, Respiratory and Critical Care Medicine, Berlin, Germany

**Keywords:** COVID-19, Post-acute COVID-19 syndrome, Fatigue, Cognitive dysfunction, Longitudinal studies

## Abstract

**Background:**

Despite the high prevalence and major disability associated with fatigue and cognitive deficits after SARS-CoV-2 infection, little is known about long-term trajectories of these sequelae. We aimed to assess long-term trajectories of these conditions and to identify risk factors for non-recovery.

**Methods:**

We analyzed longitudinal data from the population-based COVIDOM/NAPKON-POP cohort in Germany. Participants with confirmed SARS-CoV-2 infection were assessed at least 6 months (baseline) and again at least 18 months (follow-up) after infection using the Functional Assessment of Chronic Illness Therapy-Fatigue (FACIT-Fatigue) Scale (cutoff ≤ 30) and the Montreal Cognitive Assessment (MoCA, cutoff ≤ 25). Predictors of recovery from fatigue or cognitive deficits between assessments were identified through univariate and multivariable logistic regression models. The COVIDOM study is registered at the German registry for clinical studies (DRKS00023742) and at ClinicalTrials.gov (NCT04679584).

**Findings:**

Between 15 November 2020 and 9 May 2023, a total of 3038 participants were assessed at baseline (median 9 months after infection) and 83% responded to invitations for follow-up (median 26 months after infection). At baseline, 21% (95% confidence interval (CI) [20%, 23%]) had fatigue and 23% (95% CI [22%, 25%]) had cognitive deficits according to cutoff scores on the FACIT-Fatigue or MoCA. Participants with clinically relevant fatigue (at baseline) showed significant improvement in fatigue scores at follow-up (Hedges’ g [95% CI] = 0.73 [0.60, 0.87]) and 46% (95% CI [41%, 50%]) had recovered from fatigue. Participants with cognitive deficits showed a significant improvement in cognitive scores (g [95% CI] = 1.12 [0.90, 1.33]) and 57% (95% CI [50%, 64%]) had recovered from cognitive deficits. Patients with fatigue exhibiting a higher depressive symptom burden and/or headache at baseline were significantly less likely to recover. Significant risk factors for cognitive non-recovery were male sex, older age and <12 years of school education. Importantly, SARS-CoV-2 reinfection had no significant impact on recovery from fatigue or cognitive deficits.

**Interpretation:**

Fatigue and cognitive deficits are common sequelae after SARS-CoV-2 infection. These syndromes improved over time and about half of the patients recovered within two years. The identified risk factors for non-recovery from fatigue and cognitive deficits could play an important role in shaping targeted strategies for treatment and prevention.

**Funding:**

Funded by the German 10.13039/501100002347Federal Ministry of Education and Research (BMBF; grant number 01KX2121) and 10.13039/501100001659German Research Foundation (DFG) Excellence Cluster “Position Medicine in Information”.

**Research in context**

**Evidence before this study**

A literature search on PubMed of articles published between 1 March 2020 and 30 September 2023 using the search terms “fatigue” and “cognitive” as well as “LitCLONGCOVID [filter]” was conducted before data analysis. Fatigue and cognitive deficits are common sequelae of COVID-19. Results on the long-term trajectories of these two syndromes were inconclusive with some studies suggesting persistent symptoms for one to two years while others indicate decreasing symptom burden over time. Existing studies lacked comprehensive analyses of recovery predictors in affected patients.

**Added value of this study**

Utilizing validated questionnaires and in-person assessments, our study revealed significant improvements in both fatigue and cognitive deficits within the first two years post-infection. About half the patients with either syndrome recovered. Notably, persistent fatigue was predicted by baseline depression symptoms and headache, emphasizing the importance of addressing neurological and mental health factors. Non-recovery from cognitive deficits was predicted by pre-infection sociodemographic factors, but not by COVID-specific factors.

**Implications of all the available evidence**

Post-COVID fatigue and cognitive deficits improve over time, yet about 50% of patients experience symptoms that persist for up to two years. Addressing comorbid depression and headache is an important part of fatigue management. Further research is needed to deepen our understanding of the connection between SARS-CoV-2 infection and lasting cognitive deficits.

## Introduction

Three years into the COVID-19 pandemic, long-COVID has become a major health issue, with an estimated 65 million long-COVID patients globally.^1^ Fatigue and cognitive deficits are amongst the most common sequelae, affecting approximately 19% and 26% of those infected with SARS-CoV-2, respectively.^2^ These sequelae are also among the most debilitating conditions and are associated with decreased quality of life and social participation, and delayed return to work.^3^^,^^4^

However, little is known about the long-term trajectories of these sequelae. Electronic health record data suggest that the incidence of cognitive impairment and dementia remains increased during the first two years after infection, compared to both healthy controls and patients with other respiratory infections.^5^^,^^6^ However, these data often fail to capture fatigue, as it is known to be underdiagnosed and uncertainty exists about which diagnosis to encode in health records.

Most of the available data stem from previously hospitalized patients. One study found that fatigue prevalence increased from 22% to 34% during the first 12 months after hospital discharge,^7^ while another study showed a decrease in fatigue between 4 and 24 months after discharge.^8^ However, hospitalized COVID-19 patients tend to be substantially older than non-hospitalized patients, suffer from more severe disease courses, often have relevant pre-infection comorbidities, and many suffer from treatment-related side effects or complications.^9^ Findings from hospital cohorts therefore cannot be extrapolated to the majority of COVID patients who do not require hospitalization.

Population-based studies using validated instruments are better suited to accurately depict how post-COVID fatigue and cognitive deficits develop over time in both hospitalized and non-hospitalized patients. Longitudinal population-based reports, however, remain scarce. Ballouz and colleagues assessed symptom trajectories in a large Swiss cohort and found that fatigue and post-exertional malaise decreased in prevalence from 6 to 12 months after infection, but then remained stable at about 15% and 12% respectively.^10^ Interestingly, the proportion of patients scoring above the cutoff for fatigue on the Fatigue Assessment Scale remained stable around 38% between 6 and 24 months after infection. A recent study in a UK cohort found small but significant decreases in cognitive accuracy that persisted for almost two years after infection.^11^

In addition, the identification of risk factors contributing to the persistence of fatigue and cognitive deficits is critical to guide health care procedures and generate hypotheses for research on the underlying mechanisms of these syndromes. Here, again, data are scarce and adequately powered analyses are only available from inpatient cohorts,^12^ not representing the majority of patients with post-COVID fatigue and cognitive deficits.

Given the high prevalence of post-COVID fatigue and cognitive deficits,^2^ we aimed to assess long-term trajectories of these sequelae at least 18 months after infection in over 3000 patients of the population-based platform of the German National Pandemic Cohort Network (NAPKON-POP). We hypothesized that a substantial proportion of patients would recover from the two conditions on long-term follow-up. Furthermore, we aimed to identify risk factors for non-recovery from post-COVID fatigue or cognitive deficits, given the medical need to predict recovery vs. persistence in the patients.

## Methods

### Study design and participants

The COVIDOM/NAPKON-POP study is a prospective, longitudinal, population-based multicenter study in Germany. A study protocol including a sample size calculation and an analysis of sample characteristics have been published previously.^13^^,^^14^

The study included individuals with a first-time positive polymerase chain reaction (PCR) test for SARS-CoV-2 who were at least 18 years old and lived in the administrative regions of Berlin-Neukölln, Schleswig–Holstein (Kiel region) or Lower Franconia, Germany. Patients were invited to participate in the study by mail through public health authorities. Individuals who were experiencing a reinfection at baseline were excluded from the study. The baseline assessment was carried out on site at least 6 months after infection and analysis includes all participants with baseline assessment between 15 November 2020 and 9 May 2023 whose data had passed quality control at the time of data analysis.

Follow-up assessments were conducted at least 18 months after infection. Of the baseline sample, all participants received an online follow-up survey including the Functional Assessment of Chronic Illness Therapy-Fatigue (FACIT-Fatigue) questionnaire and approximately 30% were invited for an on-site appointment where the Montreal Cognitive Assessment (MoCA) was administered. The in-person appointments were assigned to cases of likely post-COVID syndrome and matched controls. Cases were defined as Post-COVID Syndrome (PCS) score ≥26.3 at baseline and controls as participants with PCS scores below this empirically determined cutoff.^15^ On-site controls were matched 1:1 based on the date of their SARS-CoV-2 PCR test: The participant whose PCR test was next in time to a defined case was selected as a control participant. If several subjects fulfilled this criterion, one of them was selected at random. Where more appointments were available, additional controls were matched to randomly selected cases until 30% of the baseline sample had been invited for on-site follow-up appointments.

### Ethics and study registration

All participants provided written informed consent in accordance with the Declaration of Helsinki. The study was approved by the responsible ethics committees (reference numbers: Berlin EA1/316/21, Kiel D537/20, Würzburg 236/20_z-am). The COVIDOM study is registered at the German registry for clinical studies (DRKS00023742) and at ClinicalTrials.gov (NCT04679584).

### Measures

#### Primary measures

The level of fatigue was measured using the FACIT-Fatigue scale, a validated questionnaire that assesses 13 fatigue symptoms on a five-point Likert scale. The total score ranges from 0 (worst fatigue) to 52 (no fatigue), with scores of 30 or lower indicating clinically significant fatigue based on general population data.^16^ Patients who scored below this cutoff at baseline and above at follow-up were considered to have recovered from fatigue. Fatigue severity was further categorized according to percentiles from German normative data as moderate (FACIT-Fatigue ≤ 30), moderately severe (FACIT-Fatigue ≤ 24, 4th percentile) and severe (FACIT-Fatigue ≤ 18, 2nd percentile).^17^ We considered a change of 3 points to be the minimal clinically important difference, since it is the established threshold in hematological and oncological populations.^18^

Cognitive performance was assessed using the MoCA, a validated screening tool that provides a total score ranging from 0 (severe cognitive deficits) to 30 (no cognitive deficits). In accordance with the testing manual, one point was added to the scores of individuals with less than 12 years of education, and an alternative version was used during follow-up to reduce learning effects. Scores ≥26 were considered normal, and 18–25 as mild, 10–17 as moderate and ≤9 as severe cognitive deficits.^19^ Patients with scores <26 at baseline and ≥26 at follow-up were considered to have recovered from cognitive deficits. A score change of ≥1.22 (anchor-based) or ≥2.15 points (distribution based) can be considered as a clinically important change, as was empirically determined in stroke survivors.^20^

#### Other measures

Information about sociodemographic, lifestyle, and clinical characteristics were gathered with a standardized questionnaire. Presence of 23 typical acute COVID symptoms and pre-infection medical diagnoses were retrieved in a standardized clinical interview and collated with medical records. The PHQ-8 was used to evaluate depressive symptoms,^21^ the GAD-7 for anxiety.^22^

### Statistical procedures

All statistical tests were two-tailed and performed in R version 4.0.2. P-values < 0.05 were considered statistically significant. Hedges’ g was used to assess effect size and scores from baseline and follow-up assessments were compared using paired t-tests.

To assess predictors of recovery, we ran separate univariate and multivariate logistic regression models containing the following independent variables: sex (male, female), age (per 5 years), school education (<12 years, ≥12 years), employment (employed, not employed), pre-infection comorbidities [any neuropsychiatric diagnosis (yes, no), anxiety disorder (yes, no), depression disorder (yes, no), sleep apnea (yes, no), chronic kidney disease (yes, no), malignant tumor disease (yes, no), cardiovascular disease (yes, no)], hospitalization during acute COVID (yes, no), number of acute COVID symptoms (range 1–23), reinfection with SARS-CoV-2 between baseline and follow-up (yes, no), depressive symptoms (PHQ-8 sum score), anxiety symptoms (GAD-7 sum score), headache (dichotomized as none/mild vs. moderate/severe), and MoCA cognitive score or FACIT-Fatigue score, respectively. Multivariable logistic regression was performed using the best subset method in R package “bestglm” and the model with the lowest Akaike information criterion (AIC) was chosen as the final model. Multicollinearity was assessed using the variance inflation factor and linearity of the logit was checked for continuous variables. Nagelkerke’s R^2^ was used to estimate goodness of model fit for the final multivariable models.

### Role of the funding source

The funders were not involved in study design, data collection, data analysis, interpretation of data, writing of the report or decision to submit the paper for publication. TJH and CF had access to the data and are finally responsible for the decision to submit the current work for publication.

## Results

### Participants

Overall, 3038 out of 3559 participants fulfilled the inclusion criteria at baseline and there were n = 2092 with at complete FACIT-Fatigue questionnaire and n = 889 with complete MoCA at follow-up (Supplementary Materials, eFig. S1). The study cohort had a median age of 44 (quartiles 31, 56) years, and median time since infection was 9 (quartiles 7, 12) months at baseline and 26 (quartiles 22, 29) months at follow-up. Further baseline characteristics are presented in Table 1 and extended cohort characteristics including number of missing values are shown in the Supplementary Materials (eTable S1).

**Table 1:** Sample characteristics at baseline.

Characteristic	N = 3,038^a^
Sociodemographic characteristics	
Sex	
Female	1694 (56%)
Male	1343 (44%)
Age [years]	
18–34	971 (32%)
35–49	834 (27%)
50–64	936 (31%)
65–88	297 (10%)
Education	
<12 years	1269 (49%)
≥12 years	1326 (51%)
Not employed	519 (17%)
Partnered	2260 (80%)
Study Center	
Kiel Universitätsklinikum	2099 (69%)
Würzburg Universitätsklinikum	549 (18%)
Berlin Charité	390 (13%)
Lifestyle	
BMI	
Underweight	36 (1%)
Normal weight	1272 (42%)
Overweight	1001 (33%)
Obese	693 (23%)
Smoker	405 (14%)
Alcohol	
Never/almost never	348 (28%)
<5x per week	769 (63%)
≥5x per week	110 (9%)
Pre-infection comorbidities	
Any neuropsychiatric disease	731 (24%)
Depression disorder	337 (11%)
Anxiety disorder	94 (3%)
Other neuropsychiatric	350 (12%)
Cardiovascular disease	733 (26%)
Sleep apnea	114 (4%)
COPD	41 (1%)
Chronic kidney disease	25 (1%)
Tumor disease	38 (1%)
Clinical characteristics	
Time since infection	
6–9 months	1402 (46%)
9–12 months	1174 (39%)
≥12 months	462 (15%)
Number of acute COVID symptoms	9 (4)
Disease course	
Home isolation	2886 (95%)
General ward	115 (4%)
Intensive care	37 (1%)
Outcome assessment	
Depression symptom severity (PHQ-8)	
None/minimal (0–4)	1528 (51%)
Mild (5–9)	943 (32%)
Moderate (10–14)	353 (12%)
Moderately severe (15–19)	126 (4%)
Severe (20–24)	20 (1%)
Anxiety symptom severity (GAD-7)	
None/minimal (0–4)	2045 (69%)
Mild (5–9)	681 (23%)
Moderate (10–14)	182 (6%)
Severe (15–21)	76 (3%)
Headache	
None	1860 (64%)
Mild	649 (22%)
Moderate	293 (10%)
Severe	114 (4%)

For extended characteristics and missing values see Supplementary Materials, eTable S1.

BMI: body mass index, COPD: chronic obstructive pulmonary disease.

a n (%); Mean (SD).

In the whole cohort, 18% were not employed at baseline and 20% at follow-up. Among patients with fatigue, 9% were not employed at baseline and 10% at follow-up. Among patients with cognitive deficits, 17% were not employed at baseline and 16% at follow-up.

### Non-responder analyses

At the time of data analysis, 71% (2154/3038) of baseline participants were due for follow-up. Out of these, 17% (373/2154) had not yet responded at the time of data export. Responders were significantly older than non-responders. There were no significant differences between responders and non-responders for any other assessed baseline characteristics (Supplementary Materials, eTable S2).

### Frequencies of fatigue and cognitive deficits

**Fig. 1:**
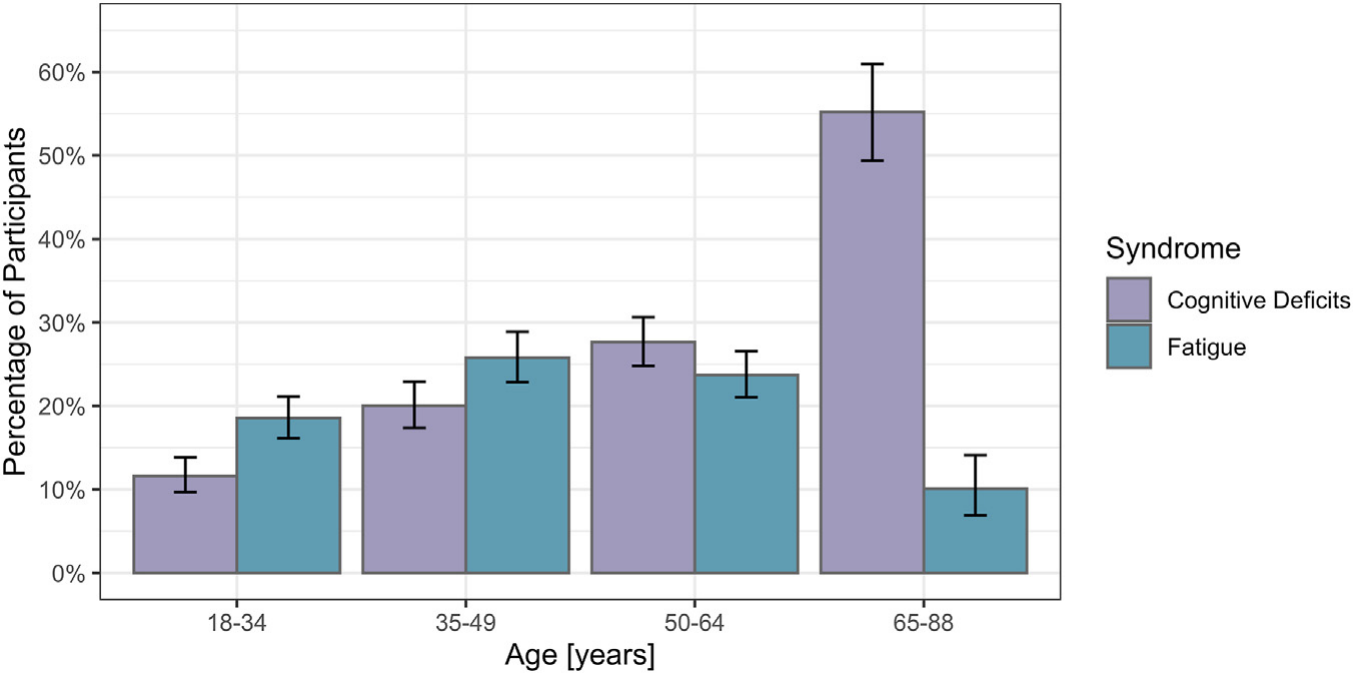
Frequency of fatigue and cognitive deficits by age group at baseline (median 9 months after infection, n = 3038). Error bars represent 95% confidence intervals.

At baseline, 21% (95% confidence interval (CI) [20%, 23%]) of participants had fatigue (9% moderate, 6% moderately severe and 6% severe fatigue). Fatigue was most common among middle-aged patients (Fig. 1). Twenty-three percent (95% CI [22%, 25%]) had cognitive deficits (22% mild, <1% moderate and <1% severe cognitive deficits). Participants in the oldest age group were most often affected by cognitive deficits. Six percent (95% CI [5%, 7%]) had both fatigue and cognitive deficits (compared to 15% with fatigue only and 17% with cognitive deficits only).

### Trajectories of fatigue and cognitive deficits

**Fig. 2:**
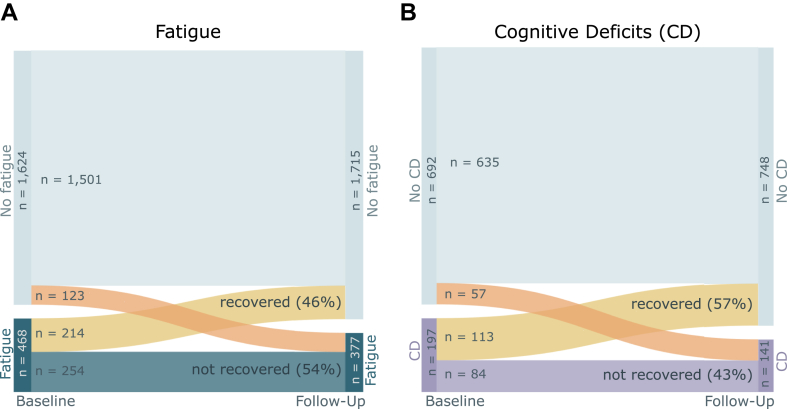
Sankey diagram of (A) fatigue (dark blue) and (B) cognitive deficits (CD; purple) at baseline (median 9 months after infection) and follow-up (median 26 months after infection). Between baseline and follow-up, 46% of patients with fatigue and 57% of patients with CD had recovered (yellow). Among patients with no fatigue/CD at baseline, 8% had developed fatigue and 9% had developed CD at follow-up (orange).

Among patients with fatigue at baseline for whom longitudinal data were available, 46% (95% CI [41%, 50%]) had recovered at follow-up, and 57% (95% CI [50%, 64%]) of patients with cognitive deficits had recovered (Fig. 2).

**Fig. 3:**
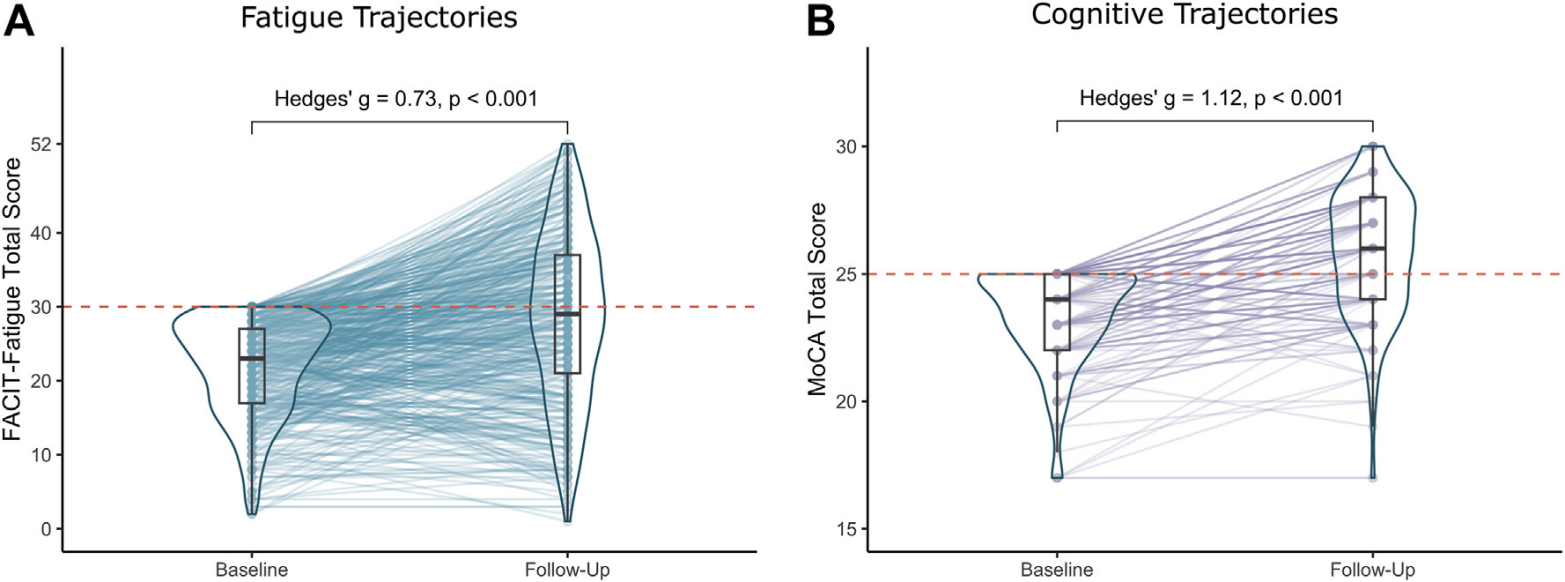
Longitudinal change in (A) FACIT-Fatigue scores for patients with fatigue at baseline (n = 468), (B) MoCA scores for patients with cognitive deficits at baseline (n = 197).

FACIT-Fatigue scores for patients with fatigue at baseline (mean 21.77, SD 6.62) had significantly improved at follow-up (mean 28.63, SD 11.45), exhibiting a large effect size (Hedges’ g [95% CI] = 0.73 [0.60, 0.87]; Fig. 3A). The mean change in FACIT-Fatigue scores (mean 6.86, 95% CI [5.66, 8.06] points) was significantly above the minimal clinically important difference of 3.00 points. MoCA scores for patients with cognitive deficits at baseline (mean 23.30, SD 1.87) had significantly improved at follow-up (mean 25.75, SD 2.46) with a large effect size (Hedges’ g [95% CI] = 1.12 [0.90, 1.33]). The mean change in MoCA scores (mean 2.45, 95% CI [2.02, 2.88] points) exceeded the conservative threshold for a minimal clinically important difference of 2.15 points and was significantly above the more liberal threshold of 1.22. For the whole cohort, there was also a significant improvement in both FACIT-Fatigue scale (mean (SD) at baseline: 38.52 (10.75), at follow-up: 39.85 (10.53), Hedges’ g [95% CI] = 0.13 [0.06, 0.19]; Supplementary Materials, eFig. S2A) and MoCA scores (mean (SD) at baseline: 26.95 (2.42), at follow-up: 27.47 (2.24), Hedges’ g [95% CI] = 0.22 [0.13, 0.32]; Supplementary Materials, eFig. S2B).

### Risk factors for non-recovery

**Fig. 4:**
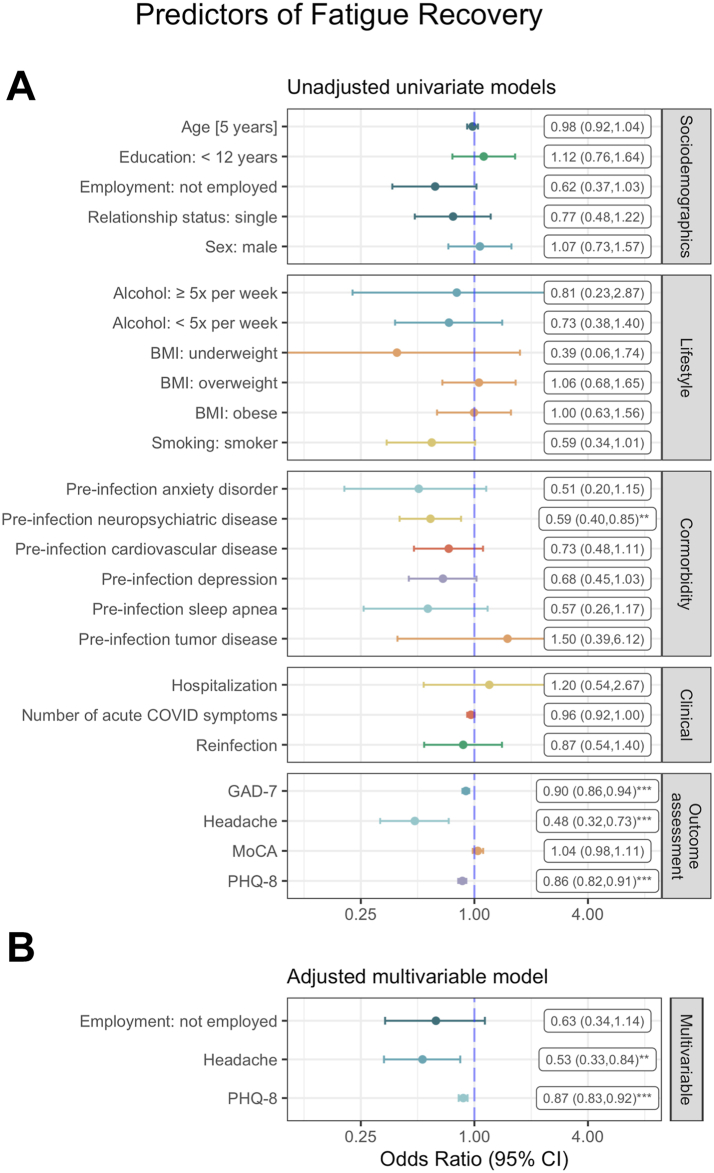
Forest plot of (A) unadjusted univariate and (B) adjusted multivariable logistic regression models for predictors of fatigue recovery (Nagelkerke’s R^2^ = 0.14). ∗p < 0.05, ∗∗p < 0.01, ∗∗∗p < 0.001.

In univariate models, pre-infection neuropsychiatric disease, depressive symptoms (PHQ-8), anxiety symptoms (GAD-7), and headache at baseline were significantly associated with non-recovery from fatigue at follow-up (Fig. 4A). The best-fitting multivariable model (AIC = 505) contained employment status, headache and PHQ-8 as independent variables, and headache as well as PHQ-8 score were independently associated with fatigue (non-)recovery (Fig. 4B). Nagelkerke’s R^2^ was 0.14.

Since GAD-7 (anxiety) and PHQ-8 (depression) scores were associated with (non-)recovery from fatigue, we assessed univariate associations of individual anxiety and depression symptoms with fatigue recovery (Supplementary Materials, eFig. S3). Loss of energy (PHQ-8, item 1) at baseline showed a strong association with non-recovery from fatigue at follow-up. In addition, not only other fatigue-like depression symptoms, but also symptoms that do not resemble fatigue such as anticipatory fear (GAD-7, item 7) were significantly associated with non-recovery from fatigue.

**Fig. 5:**
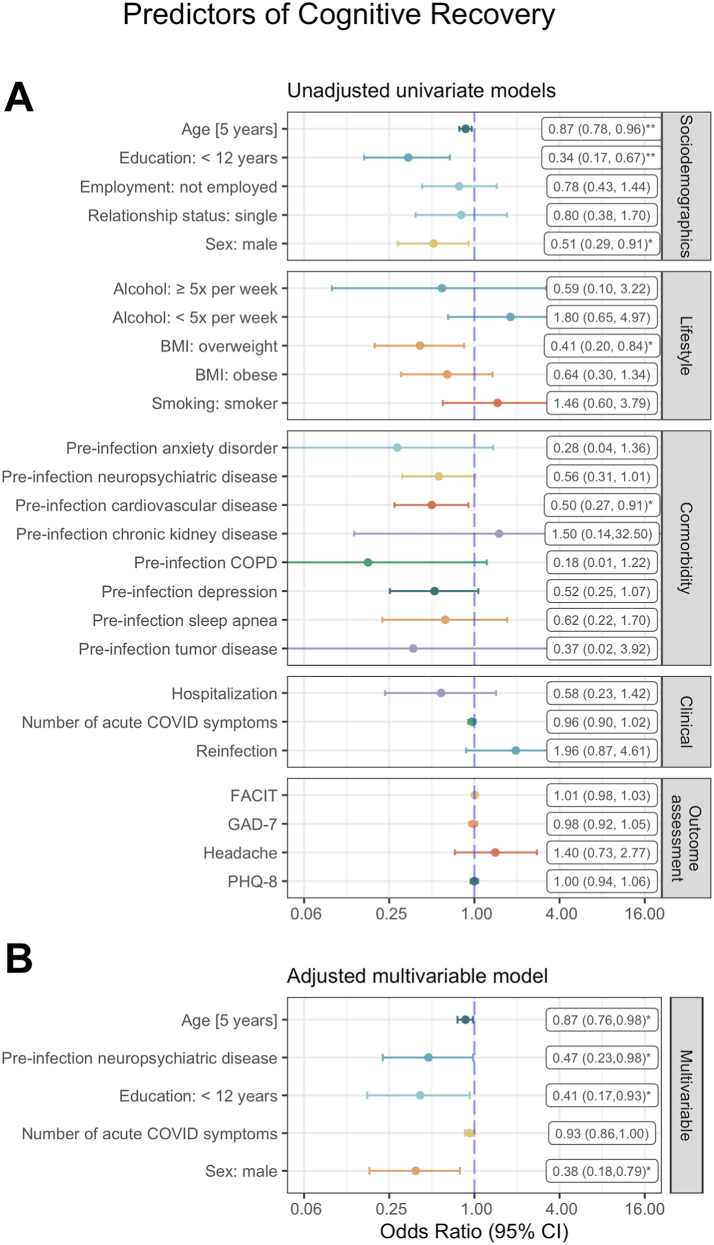
Forest plot of (A) unadjusted univariate and (B) adjusted multivariable logistic regression models for predictors of cognitive recovery (Nagelkerke’s R^2^ = 0.19). ∗p < 0.05, ∗∗p < 0.01, ∗∗∗p < 0.001.

In univariate models of cognitive recovery, sex, age, education, BMI, and pre-infection cardiovascular disease at baseline showed significant associations with cognitive (non-)recovery (Fig. 5A). The best-fitting (AIC = 208) multivariable model of cognitive recovery contained sex, age, education, pre-infection neuropsychiatric comorbidity and number of acute COVID symptoms as independent variables (Fig. 5B). According to the multivariable model, patients with male sex, older age and school education <12 years had significantly lower odds to recover from cognitive deficits (Fig. 5B). Nagelkerke’s R^2^ was 0.19.

## Discussion

In this population-based longitudinal study, we assessed fatigue and cognitive deficits using validated instruments with a median follow-up duration of 26 months since infection. We observed that half of the patients with post-COVID fatigue and/or cognitive deficits recovered within the follow-up period. Risk factors for non-recovery from fatigue were headache and depressive symptom burden, whereas non-recovery from cognitive deficits was predicted by older age, male sex and less than 12 years of school education. Our results provide important insights into the longitudinal trajectory of fatigue and cognitive deficits following COVID-19 that allow to estimate the long-term disease burden associated with post-COVID syndrome and help to develop tailored care and rehabilitation programs.

At baseline (i.e., at median 9 months after infection), 21% of participants reported clinically relevant fatigue, including 6% of participants with moderately severe and another 6% with severe levels of fatigue. This suggests a much higher burden of fatigue symptoms after SARS-CoV-2 infection than in the pre-pandemic general population where 9% reported clinically relevant fatigue, including 2% with moderately severe and another 2% with severe levels.^17^ Frequency and age distribution of fatigue were highly consistent with our findings in the first 1000 participants from this cohort.^2^ At follow-up (median 26 months after infection), fatigue scores of patients with post-COVID fatigue improved significantly and with a large effect size (g = 0.73). The mean change in FACIT-Fatigue scores was significantly above the threshold for a clinically important change.^18^ Indeed, findings from health record data and hospital records also suggest a decrease in incidence of fatigue-like diagnoses and fatigue symptoms.^6^^,^^8^ However, reliable long-term assessments in non-hospitalized patients were not available so far. Here, we show that approximately half of the patients with post-COVID fatigue recover within about two years after infection. This suggests that fatigue symptoms can resolve in many patients even though causal treatments are not yet available. At the same time, our data show that half of the patients with fatigue suffer from long-term persisting symptoms with potentially detrimental effects on quality of life, ability to work, and social and mental wellbeing.

Independent predictors of persisting fatigue at follow-up were depressive symptom burden and headache at baseline. Indeed, our cross-sectional analysis from this cohort already identified pre-infection depression disorder as a significant predictor of post-COVID fatigue.^2^ In a recent cohort study, pre-infection psychological distress was also associated with an increased risk for long-COVID conditions.^23^ This suggests that psychosocial factors may at least contribute to the persistence of these symptoms and their impact on patients’ quality of life. A recently conducted randomized controlled trial indicated that antidepressant medication can alleviate depression symptoms in patients with post-COVID syndrome.^24^ It is crucial to investigate whether targeted diagnosis and treatment of psychosocial distress in general, and depression in particular, can also improve long-term fatigue in these patients.

Headache is one of the most common sequelae of COVID-19.^25^^,^^26^ Indeed, we found that 22% of patients reported mild headache and 14% moderate to severe headache at baseline (median 9 months after infection). A study using smartphone-based self report also found that headache during the acute stage of COVID-19 was a risk factor for the development of long-COVID and was strongly associated with fatigue.^26^ We now show that moderate to severe headache during the post-COVID period is an independent risk factor for persisting fatigue beyond 18 months after infection. Future studies should investigate potential common mechanisms of headache and fatigue in this population and evaluate if timely diagnosis and treatment of post-COVID headache has an impact on long-term fatigue.

Remarkably, our models on risk factors for persisting long-term fatigue explained only a relatively small proportion of the variance in fatigue recovery (Nagelkerke’s R^2^ = 0.14). This is despite the fact that these models included a broad spectrum of detailed data on sociodemographic characteristics, lifestyle, comorbidity, clinical parameters and psychological factors. Interestingly, reinfection with SARS-CoV-2 had no relevant impact on fatigue recovery. This suggests that either the phenotype or the risk factors for post-COVID fatigue are more complex than previously thought. On the one hand, the current conceptualizations of fatigue may not represent the heterogeneous group of patients with post-COVID fatigue. Future studies should further investigate the phenotype and potential sub-types of this syndrome or characterize it on a symptom level, assessing constellations, trajectories and interactions of individual symptoms. On the other hand, the associated risk factors may not contribute to the pathophysiology in isolation but may rather constitute a more complex interplay of biological, social and psychological factors. Recently, we found reduced microstructural integrity of the basal ganglia and the thalamus in patients with post-COVID fatigue, which was also correlated with the severity of fatigue symptoms.^27^ Future studies should investigate such structural and functional brain changes and other biomarkers as well as psychosocial factors in an integrated bio-psycho-social approach.

About 23% of participants had cognitive deficits at baseline. Remarkably, even among younger participants under 35 years, 12% (113/971) were affected. At follow-up, patients with post-COVID cognitive deficits showed large improvements, and about half had recovered within the follow-up period. The mean change in MoCA scores was also above the threshold for clinically important change.^20^ This shows that cognitive deficits can be transient in a large proportion of patients, and these patients likely experience little to no limitations in their activities of daily life. At the same time, our results indicate that cognitive deficits may persist in a relevant subset of patients.

The high rate of spontaneous cognitive recovery (57%) is in contrast to electronic health record data which showed a persistently increased incidence of cognitive sequelae of COVID-19 during the first two years after infection.^6^ However, health records contain information on cognitive deficits and dementia only if the severity of such deficits mandated medical help. Further, diagnostic assessments are usually only performed in selected groups of patients. Our study, to the contrary, used a sensitive screening instrument in a population-based sample and was able to detect more subtle, yet clinically relevant, cognitive deficits. Also, the incidence of newly diagnosed cognitive deficits provides little information on trajectories over time and does not allow conclusions on temporal let alone causal relationships, since every patient is only depicted once in incidence data. Our results therefore complement incidence data from registries by depicting the long-term trajectory of the whole spectrum of cognitive deficits.

While the MoCA is an excellent screening tool for cognitive deficits, it does not offer the same diagnostic accuracy as a detailed neuropsychological assessment. A recent study in patients after COVID-19 found that the MoCA had a sensitivity of only 50% for cognitive deficits detected using a comprehensive cognitive test battery, while specificity (83%) and positive predictive value (82%) were relatively high.^28^ This suggests that subtle cognitive deficits may not be detected by the MoCA in patients after COVID and that the frequency of cognitive deficits in our study may underestimate the actual prevalence. Future studies should therefore assess post-COVID cognitive deficits with more detailed neuropsychological assessments.

Risk factors for non-recovery from cognitive deficits were older age, male sex, and less than 12 years of school education. As such, pre-infection characteristics best predicted cognitive non-recovery and no COVID-specific risk factors showed significant associations. This suggests that cognitive deficits largely depend on pre-existing general factors. In many cases, COVID-19 may unmask or exacerbate neurodegenerative processes that could already be ongoing before infection. At the same time, it is crucial to investigate in more detail additional factors that contribute to the development and persistence of cognitive deficits. These may include biological factors such as serum markers of inflammation or structural and functional brain changes that can be investigated with neuroimaging.

Notably, only 6% suffered from both fatigue and cognitive deficits, which is consistent with earlier results from the first 1000 participants of this cohort.^2^ This suggests that the two syndromes are distinct in terms of who is affected and potentially also in their underlying pathophysiological mechanisms. Interestingly, a similar proportion improved and score improvements showed similarly large effect sizes in the two patient groups. This suggests a similar degree of reversibility for symptoms of fatigue and cognitive deficits.

Given that there are over 275 million confirmed cases of COVID-19 in Europe alone, an extrapolation from our findings would suggest that up to 100 million of them may suffer from clinically relevant levels of fatigue, cognitive deficits or both, 6–12 months after infection.^29^ Two years after infection, there may still be up to 50 million affected by these syndromes in Europe. This situation has recently been described as a “burgeoning public health crisis” and may entail that millions of people could not return to work, suffer from a reduced quality of life and impaired social participation.^30^ Hence, there is an urgent need to better understand the pathophysiological mechanisms involved and to develop effective therapies.

Strengths of the study include the large, population-based sample, longitudinal study design and use of validated instruments. Previous analyses showed that the cohort was representative of the general population in all major sociodemographic characteristics.^14^ Clinical characteristics such as the hospitalization rate also mirrored those of the German population at the time.^2^^,^^31^ An additional in-depth analysis for the Schleswig–Holstein region showed that the cohort was highly representative of the infected population across almost all adult age groups.^15^ Our detailed non-responder analyses demonstrated a high response rate and showed no indication of response bias in terms of any assessed baseline characteristics except age (Supplementary Materials, eTable S2).

The generalizability of our findings is limited by the following factors: (1) The invitation strategy for on-site follow-up appointments was designed to include all cases of likely post-COVID syndrome. While fatigue was assessed in all participants at follow-up and should be highly representative, frequencies of cognitive deficits at follow-up may be biased by the design and should therefore not be interpreted as prevalence estimates. (2) Although an alternative version of the MoCA was used at follow-up, small learning effects may have contributed to the increase in MoCA scores at follow-up. (3) The FACIT-Fatigue questionnaire assesses the severity of general fatigue symptoms. Clinically relevant levels of fatigue according to the FACIT-Fatigue questionnaire, however, do not necessarily correspond to the presence of a specific fatigue syndrome such as myalgic encephalomyelitis/chronic fatigue syndrome (ME/CFS). (4) Some of the improvement in FACIT-Fatigue and/or MoCA scores in affected individuals may be due to regression to the mean. However, there was also a significant improvement in the entire cohort, suggesting that the effect is not entirely explained by regression to the mean. In addition, even if regression to the mean contributes to improving trajectories, this still suggests clinically relevant and meaningful improvements for a large number of patients with post-COVID fatigue and cognitive deficits.

In conclusion, both fatigue and cognitive deficits are common sequelae after SARS-CoV-2 infection, affecting 21% and 23% of patients in the first year after infection, respectively. Our longitudinal analyses show that the severity of these syndromes improves over time, and about half of the patients with either syndrome recover within about 2 years. However, half of the patients experience persistence of these syndromes, posing a considerable challenge for public health systems. While sociodemographic, neurological and psychological risk factors may contribute to persistence of fatigue and cognitive deficits, more research is needed to better understand the underlying neurological mechanisms that cause long-term morbidity and to develop effective treatments.

## Contributors

Conceptualization: TJH, TB, WM, CF.

Data curation: TJH, ICS, KF, JG, AH, C Maetzler, FAM, C Nürnberger, C Neumann, AKR, LS, FS.

Formal analysis: TJH, FS, CF.

Funding acquisition: TB, PUH, TK, MK, WL, S Schreiber, TZ, WM, CF.

Investigation: TB, JG, AH, TK, C Maetzler, FAM, C Morbach, C Nürnberger, C Neumann, AKR, TZ.

Methodology: TJH, TB, KF, MK, AKR, WM, S Schmidt, CF, S Schreiber.

Project administration: TB, ICS, JD, ME, KGH, PUH, SMP, AH, MK, S Schreiber LK, WL, C Nürnberger, S Schmidt, WM, CF.

Resources: TB, JD, ME, KGH, GH, PUH, TK, MK, LK, WL, C Morbach, S Schmidt, S Schreiber, S Störk, WM, CF.

Supervision: TB, KGH, PUH, C Morbach, WM, CF.

Visualization: TJH, CF.

Writing—original draft: TJH, CF.

Writing—review & editing: TB, ICS, JD, ME, KF, JG, KGH, GH, PUH, SMP, AH, TK, MK, LK, WL, C Maetzler, FAM, C Morbach, C Nürnberger, C Neumann, AKR, LS, S Schmidt, S Schreiber, FS, S Störk, TZ, WM, CF.

## Data sharing statement

All data of this study may be shared upon request to the NAPKON data use and access committee. Interested parties can access relevant data governance information and submit their research proposal to the NAPKON use and access committee at https://proskive.napkon.de.

## Declaration of interests

TB has received grants from the German Center for Lung Research (DZL), consulting fees from AstraZeneca, GlaxoSmithKline, Pfizer, honoraria from AstraZeneca, GlaxoSmithKline, Novartis, Roche, Chiesi, Boeringer-Ingelheim, Merck, Pfizer, support for attending meetings and/or travel from Chiesi, AstraZeneca, and participated on a Data Safety Monitoring Board or Advisory Board for CoVit-2 (NCT04751604).

JD has received grants from the BMBF and participated on a Data Safety Monitoring Board or Advisory Board for the Max-Planck-Institute of Psychiatry, served as a section speaker for DGPPN, as president and treasurer for the DGBP, and as a speaker for NUM.

ME has received grants and consulting fees from Bayer and honoraria from Bayer, Pfizer, Amgen, GSK, and Novartis, participated on a Data Safety Monitoring Board or Advisory Board for BMS, Bayer, and Daiichi Sankyo, served on the board of directors for EAN. All of the above were paid to the institution (no personal fees). He is a member of the DGN, ISCBFM, AHA/ASA, ESO, WSO, DZHK (German Centre of Cardiovascular Research) and DZNE (German Center of Neurodegenerative Diseases) and has received PCSK9 inhibitors for mouse studies from Amgen.

KGH has received consulting fees from Edwards Lifesciences, Premiere Research Bayer Healthcare, Amarin, Alexion, Daiichi Sankyo. AstraZeneca, and Portola, and honoraria from Bayer Healthcare, Pfizer, Daiichi Sankyo, Bristol-Myers-Squibb, Boehringer Ingelheim, AstraZeneca, Abbott, SUN Pharma, and Novartis.

PUH has received grants from the German Ministry of Research and Education, European Union, German Parkinson Society, University Hospital Würzburg, German Heart Foundation, Federal Joint Committee (G-BA) within the Innovationfond, German Research Foundation, Bavarian State, German Cancer Aid, Charité—Universitätsmedizin Berlin (within Mondafis; supported by an unrestricted research grant to the Charité from Bayer), University Göttingen (within FIND-AF randomized; supported by an unrestricted research grant to the University Göttingen from Boehringer-Ingelheim), University Hospital Heidelberg (within RASUNOA-prime; supported by an unrestricted research grant to the University Hospital Heidelberg from Bayer, BMS, Boehringer-Ingelheim, Daiichi Sankyo). All of the above were paid to the respective institutions (no personal fees). He participated on a Data Safety Monitoring Board in publicly funded studies (by German Research Foundation, German Ministry of Research, Foundations).

FAM has received funding as part of the UNION-CVD Clinician-Scientist Programme (project number 413657723) by the German Research Foundation.

CN has received grants from Internal Medicine Department I, University Hospital Schleswig Holstein, Campus Kiel and honoraria from CAU Kiel.

CF has received grants from Deutsche Forschungsgemeinschaft (DFG, German Research Foundation), Grant numbers FI 2309/1-1 (Heisenberg Program) FI 2309/2-1 and 327654276 (SFB 1315), and the German Ministry of Education and Research (BMBF), Grant numbers 01GM1908D, 01GM2208C, 13GW0566D, 01GM2102, 01EP2201.

## Appendix ASupplementary data

Supplementary eFigs. S1–S3 and eTables S1 and S2

Extended Acknowledgements
