# Supplementary material for: Predictors of non-recovery from fatigue and cognitive deficits after COVID-19: a prospective, longitudinal, population-based study
Source: eClinicalMedicine. 2024 Feb 3;69:102456. doi: 10.1016/j.eclinm.2024.102456 (PMC10847699; doi:10.1016/j.eclinm.2024.102456)
Supplement: Supplementary eFigs. S1–S3 and eTables S1 and S2 [file mmc1.pdf]

Supplementary Materials

Predictors of recovery from fatigue and cognitive deficits after COVID-19: a prospective, longitudinal, population-based study

2023-12-14

Contents

STROBE flow chart **2**

Extended baseline characteristics **3**

Non-responder analyses **5**

Score changes in the entire cohort **6**

Psychological symptoms associated with fatigue recovery **8**

STROBE flow chart

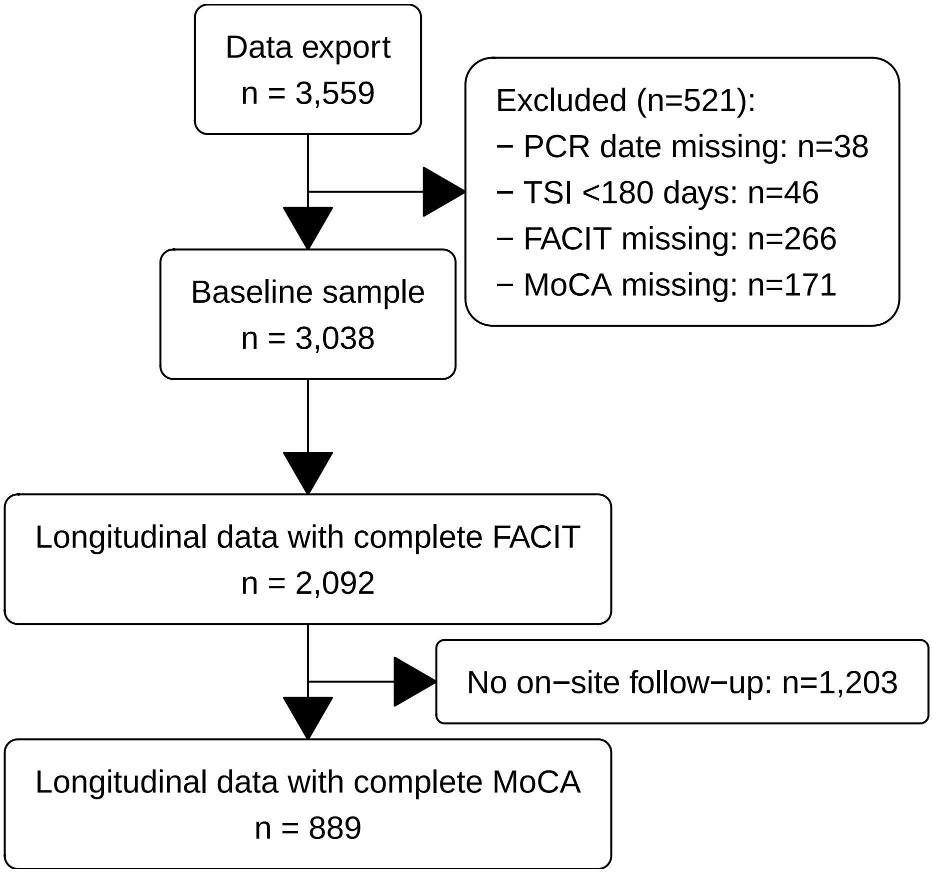

eFigure 1. STROBE flow chart for cohort studies with reasons for exclusion. Abbreviations: PCR polymerase chain reaction, TSI time since infection, FACIT Functional Assessment of Chronic Illness Therapy-Fatigue, MoCA Montreal Cognitive Assessment.

Extended baseline characteristics

eTable 1. Extended sample characteristics at baseline including missing values.

**Characteristic**	**Missing**	**N = 3,038**[1]
Sociodemographic characteristics		
Sex	1 (<0.1%)	
female		1,694 (56%)
male		1,343 (44%)
Age [years]	0 (0%)	
18-34		971 (32%)
35-49		834 (27%)
50-64		936 (31%)
65-88		297 (10%)
Education	443 (15%)	
< 12 years		1,269 (49%)
at least 12 years		1,326 (51%)
Unemployment	0 (0%)	519 (17%)
Partnered	223 (7.3%)	2,260 (80%)
Lifestyle		
BMI	36 (1.2%)	
normal weight		1,272 (42%)
obese		693 (23%)
overweight		1,001 (33%)
underweight		36 (1%)
Smoking	190 (6.3%)	
ex-smoker		1,018 (36%)
never-smoker		1,425 (50%)
smoker		405 (14%)
Alcohol	1,811 (60%)	
< 5x per week		769 (63%)
at least 5x per week		110 (9%)
Never / almost never		348 (28%)
Comorbidities		
Any neuropsychiatric disease	48 (1.6%)	731 (24%)
Depression disorder	35 (1.2%)	337 (11%)
Migraine	37 (1.2%)	278 (9%)
Anxiety disorder	19 (0.6%)	94 (3%)
Epilepsy	15 (0.5%)	22 (1%)
Ischemic stroke	22 (0.7%)	20 (1%)
Neuromuscular diseases	15 (0.5%)	13 (0%)
Multiple sclerosis	14 (0.5%)	10 (0%)
Psychotic disorder	15 (0.5%)	9 (0%)
Parkinson's disease	16 (0.5%)	4 (0%)
Hemorrhagic stroke	22 (0.7%)	4 (0%)
Dementia	14 (0.5%)	1 (0%)
Sleep apnea	82 (2.7%)	114 (4%)
COPD	51 (1.7%)	41 (1%)
Chronic kidney disease	1 (<0.1%)	25 (1%)
Cardiovascular disease	246 (8.1%)	733 (26%)

Tumor disease	11 (0.4%)	38 (1%)
Clinical characteristics		
Time since infection	0 (0%)	
6-9 months		1,402 (46%)
9-12 months		1,174 (39%)
at least 12 months		462 (15%)
Number of acute COVID symptoms [1-23]	295 (9.7%)	9 (4)
Disease course	0 (0%)	
general ward		115 (4%)
home isolation		2,886 (95%)
intensive care		37 (1%)
Neurological / psychiatric outcomes		
Depression symptom severity (PHQ-8)	68 (2.2%)	
Mild		943 (32%)
Minimal		1,528 (51%)
Moderate		353 (12%)
Moderately Severe		126 (4%)
Severe		20 (1%)
Anxiety symptom severity (GAD-7)	54 (1.8%)	
Mild		681 (23%)
Minimal		2,045 (69%)
Moderate		182 (6%)
Severe		76 (3%)
Headache	122 (4.0%)	
None		1,860 (64%)
Mild		649 (22%)
Moderate		293 (10%)
Severe		114 (4%)

[1]n (%); Mean (SD)

Non-responder analyses

At the time of data analysis, **71 %** (2154 / 3038) of baseline participants were due for follow-up.

Out of these, **17 %** (373 / 2154) had not responded at the time of data analysis.

Follow-up responders were significantly older than non-responders. There were no significant differences between responders and non-responders for any of the other assessed baseline characteristics.

eTable 2. Baseline characteristics of follow-up responders and non-responders.

Characteristic	N	**Responder**, N = 1,781	**Non-responder**, N = 373	p-value
**Sex**	2,154			0.8
female		999 (56%)	206 (55%)	
male		782 (44%)	167 (45%)	
**Age [years]**	2,154	47 (32, 58)	41 (31, 55)	**0.008**
**Education**	2,064			0.5
< 12 years		811 (47%)	158 (45%)	
at least 12 years		904 (53%)	191 (55%)	
**Any neuropsychiatric disease**	2,129	446 (25%)	88 (24%)	0.6
**Depression disorder**	2,131	215 (12%)	35 (9.5%)	0.15
**Anxiety disorder**	2,146	55 (3.1%)	15 (4.0%)	0.4
**Sleep apnea**	2,093	82 (4.7%)	10 (2.8%)	0.10
**Chronic Kidney Disease**	2,154	12 (0.7%)	1 (0.3%)	0.7
**Cardiovascular disease**	1,928	444 (28%)	92 (28%)	>0.9
**Tumor disease**	2,148	24 (1.4%)	5 (1.3%)	>0.9
**Number of acute COVID symptoms**	1,948	9 (5, 12)	9 (6, 12)	0.4
**Disease course**	2,154			0.8
general ward		84 (4.7%)	20 (5.4%)	
home isolation		1,672 (94%)	347 (93%)	
intensive care		25 (1.4%)	6 (1.6%)	
**MoCA**	2,154	27 (26, 29)	27 (25, 29)	0.8
**FACIT Fatigue**	2,154	42 (32, 47)	42 (34, 47)	0.3
**PHQ-8**	2,093	4 (2, 8)	4 (2, 7)	0.2
**GAD-7**	2,107	2 (0, 6)	2 (0, 5)	>0.9

Score changes in the entire cohort

Across all participants, FACIT-Fatigue scores significantly improved on average with a small effect size (mean (SD) at baseline: 38.52 (10.75), at follow-up: 39.85 (10.53), Hedges' g [95% CI] = 0.13 [0.06, 0.19]).

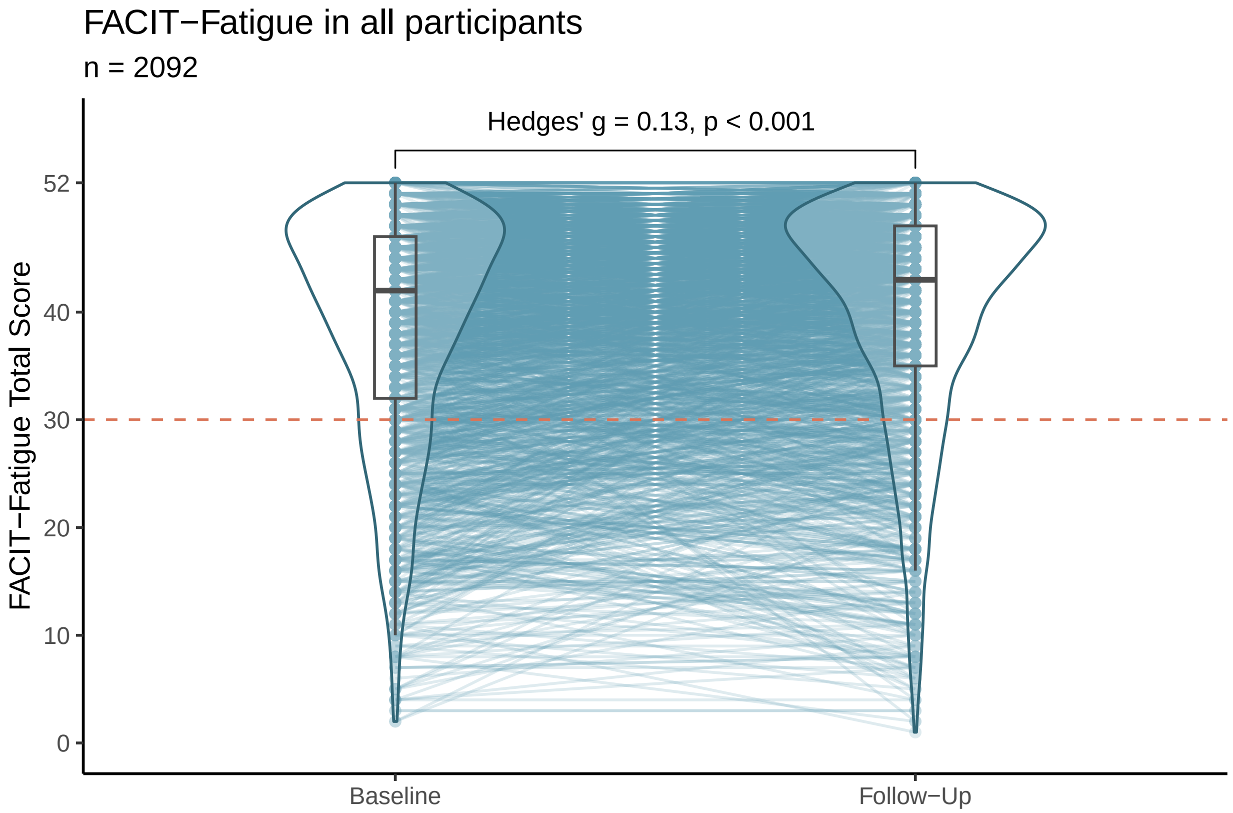

eFigure 2A. Change in FACIT-Fatigue scores in the entire cohort between baseline and follow-up.

Across the entire cohort, MoCA scores also significantly improved on average with a small effect size (mean (SD) at baseline: 26.95 (2.42), at follow-up: 27.47 (2.24), Hedges' g [95% CI] = 0.22 [0.13, 0.32]).

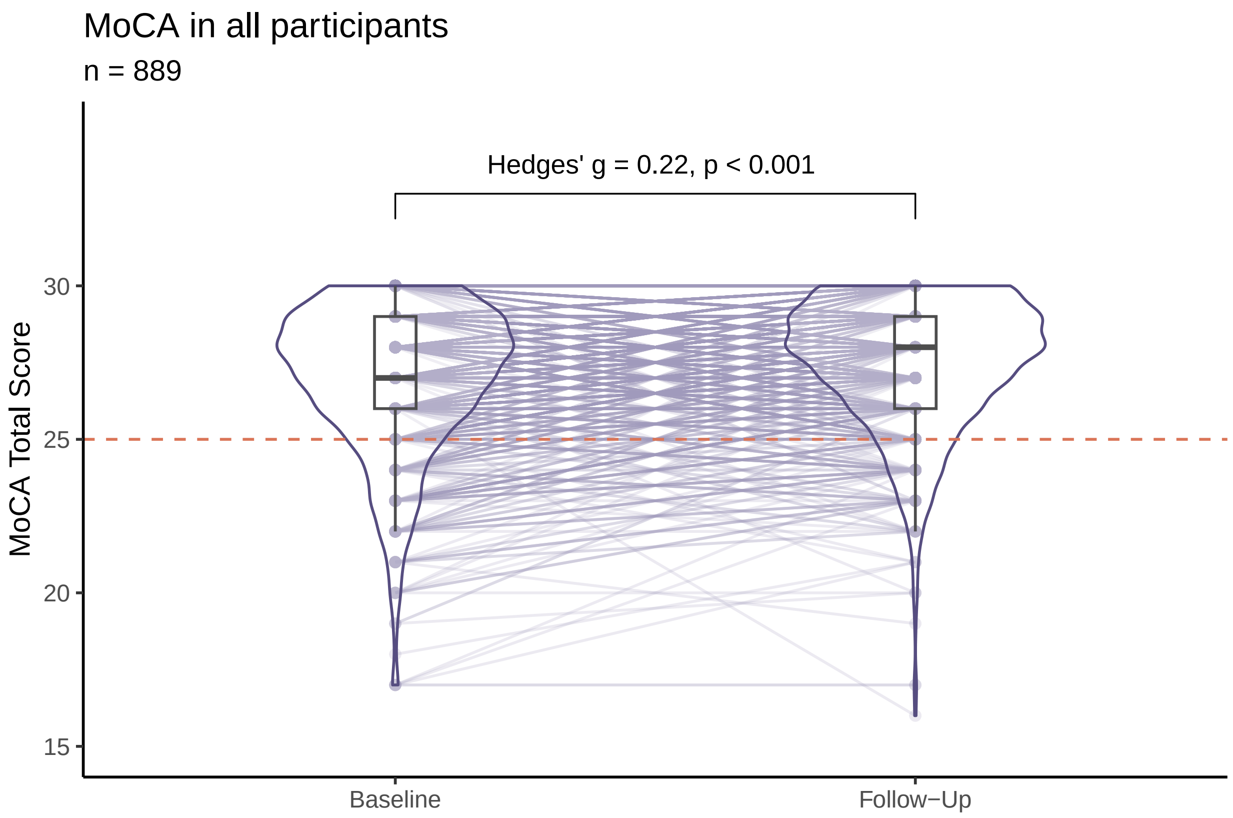

eFigure 2B. Change in MoCA cognitive scores in the entire cohort between baseline and follow-up.

Psychological symptoms associated with fatigue recovery

Depressive symptoms (PHQ-8 sum score) and anxiety (GAD-7 sum score) were significantly associated with non-recovery from fatigue. We therefore assessed which items from these questionnaires showed the strongest associations with fatigue recovery.

Loss of energy (PHQ-8, item 4) at baseline showed a strong association with non-recovery from fatigue during the follow-up period. In addition, not only other fatigue-like depression symptoms, but also symptoms that do not resemble fatigue such as anticipatory fear (GAD-7, item 7) were significantly associated with non-recovery from fatigue.

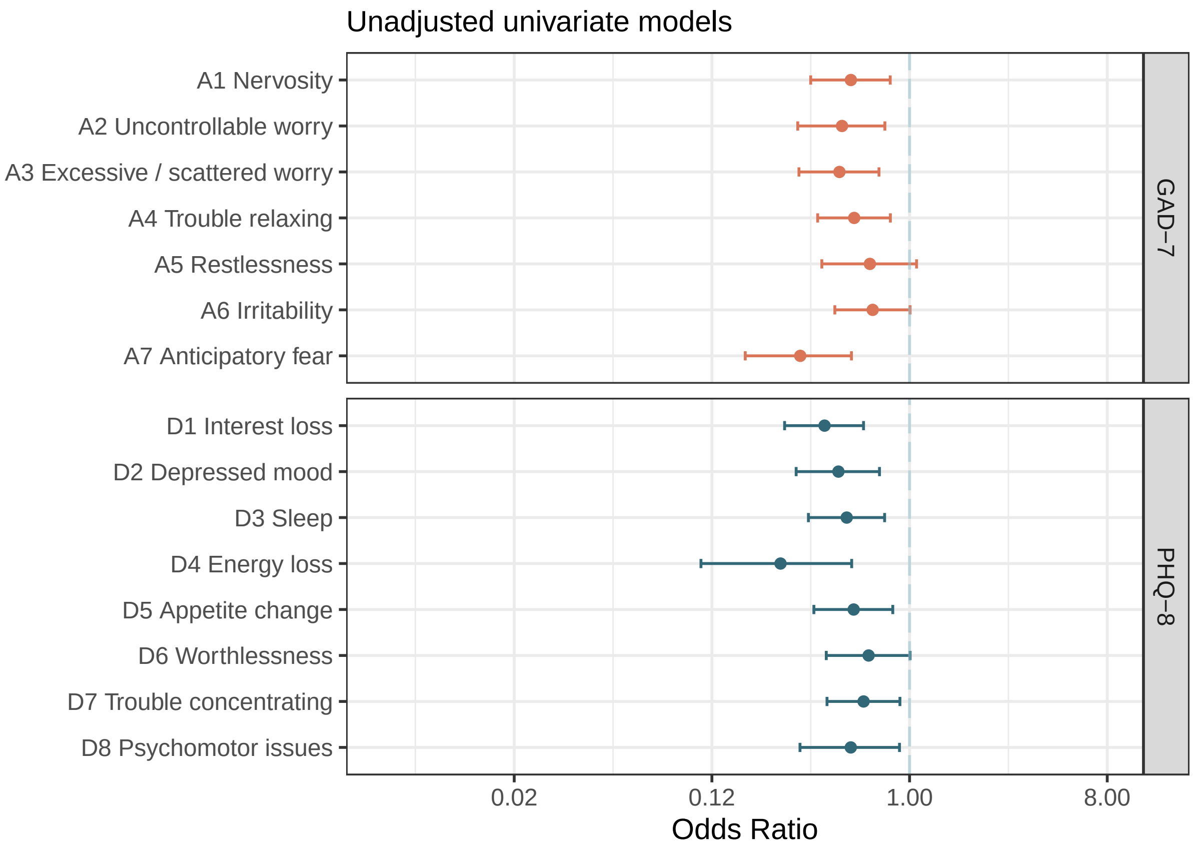

eFigure 3. Forest plot of univariate associations of anxiety (GAD-7) and depression (PHQ-8) symptoms with fatigue recovery. Error bars indicate 95% confidence interval.
