# Supplementary material for: Predictors of non-recovery from fatigue and cognitive deficits after COVID-19: a prospective, longitudinal, population-based study
Source: eClinicalMedicine. 2024 Feb 3;69:102456. doi: 10.1016/j.eclinm.2024.102456 (PMC10847699; doi:10.1016/j.eclinm.2024.102456)
Supplement: Extended Acknowledgements [file mmc2.pdf]

Extended Acknowledgements

The representatives of NAPKON sites contributing at least 5 per mille to this analysis are (alphabetical order): Charité - Universitaetsmedizin Berlin, Berlin (Fricke J, Keil T, Kretzler L, Krist L, Schmidt S, Steinbeis F, Treue D, Triller P, Witzenrath M, Zoller T), University Hospital Schleswig-Holstein, Kiel (Bahmer T, Hermes A, Krawczak M, Lehmann I, Lieb W, Maetzler C, Pape D , Reinke L, Schreiber S, Tamminga T), University Hospital Wuerzburg, Wuerzburg (Frantz S, Haeusler KG, Hein G, Horn A, Jahns R, Nürnberger C, Montellano FA, Morbach C, Stoerk S, Weissbrich B).

We gratefully thank all participating NAPKON infrastructures that contributed to this analysis. The representatives of these NAPKON infrastructures are (alphabetical order): University Hospital Cologne, Cologne (Brechtel M, Fiedler K, Hopff SM, Lee C, Nunes de Miranda S, Seibel C, Vehreschild JJ), University Hospital Frankfurt, Frankfurt (Appel KS, Geisler R, Hagen M, Schneider J, Sikdar S, Vehreschild JJ, Weismantel C, Wolf L), University of Wuerzburg, Wuerzburg (Fiessler C, Günther K, Jiru-Hillmann S, Haug F, Haug J, Heuschmann PU, Miljukov O, Nürnberger C, Reese J-P, Schmidbauer L), University Medicine Greifswald, Greifswald (Bahls T, Hoffmann W, Nauck M, Schaefer C, Schattschneider M, Stahl D, Valentin H), University Medicine Goettingen, Goettingen (Chaplinskaya I, Hanß S, Krefting D, Pape C, Rainers M, Schoneberg A, Weinert N), Helmholtz Center Munich, Munich (Kraus M, Lorenz-Depiereux B), Charité - Universitaetsmedizin Berlin, Berlin (Lorbeer R, Schaller J, Fricke J, Krist L, Rönnefarth M, Schmidt S), University Hospital Schleswig-Holstein, Kiel (Bahmer T, Hermes A, Krawczak M, Lieb W, Schreiber S, Tamminga T), University Hospital Wuerzburg, Wuerzburg (Horn A, Kohls M).

We gratefully thank the NAPKON Steering Committee: Bielefeld University, Bielefeld (Anton, G), University Hospital Frankfurt, Frankfurt (Ciesek S, Vehreschild M), University Hospital Giessen and Marburg, Giessen (Herold S), University of Wuerzburg, Wuerzburg (Heuschmann P), Charité - Universitaetsmedizin Berlin, Berlin (Heyder R, Witzenrath M), University Medicine Greifswald, Greifswald (Hoffmann W), Hannover Unified Biobank, Hannover Medical School, Hannover (Illig T), University Hospital Schleswig-Holstein, Kiel (Schreiber S), University Hospital Cologne and University Hospital Frankfurt, Cologne and Frankfurt (Vehreschild JJ).
